# Detection of Angiogenic T Cells and Endothelial Progenitor Cells in Behçet Disease and Determination of Their Relationship with Disease Activity

**DOI:** 10.3390/life13061259

**Published:** 2023-05-25

**Authors:** Ayhan Kul, Nurinnisa Ozturk, Asli Koseoglu Kurt, Yasar Arslan

**Affiliations:** 1 Faculty of Medicine, Physical Medicine and Rehabilitation, Ataturk University, Erzurum 25240, Turkey; 2 Faculty of Medicine, Medical Biochemistry, Ataturk University, Erzurum 25240, Turkeyaslikoseoglu_0808@hotmail.com (A.K.K.)

**Keywords:** Behçet disease, angiogenic T cells, endothelial progenitor cells

## Abstract

Angiogenic T (Tang) cells and endothelial progenitor cells (EPCs) play a role in maintaining vascular integrity and repair. This study considers the association between them and Behçet disease (BD) and disease activity. Fifty patients with BD and forty-five age- and gender-matched healthy controls were included in the study. The participants’ demographic, clinical, and laboratory characteristics were recorded, and their blood Tang cell and EPC counts were determined. Fifty patients were diagnosed with BD, consisting of 24 females and 26 males. The blood Tang cell (3.5 ± 1.2 cells/μL in patients, 4 ± 0.9 cells/μL in controls, *p* = 0.046)) and EPC (2.9 ± 0.9 cells/μL in patients, 3.7 ± 1 cells/μL in controls, *p* = 0.001) counts were significantly lower for the patient group with BD than for the control group. The blood Tang cell (42.5 ± 4.9% in active patients, 48.9 ± 7.9% in inactive patients, *p* = 0.001) and EPC (35.5 ± 6.4% in active patients, 41.2 ± 6.3% in inactive patients, *p* = 0.004) levels were lower for the patient group with active BD than for the inactive patient group. A weak positive correlation was present between the blood Tang cell and EPC percentage values in BD (r: 0.318, *p* = 0.002). It was determined that Tang cell and EPC counts are lower in BD, and these reductions become more profound with increasing disease activity. This situation may prevent the development of a sufficient immune response against a disease with a course of chronic inflammation or may trigger the formation of autoreactive immunity. A reduction in Tang cells and EPCs may serve as a marker or predictor of vascular damage in BD patients and represents the progression of vascular injury.

## 1. Introduction

Behçet disease (BD) is a form of vasculitis, and endothelial dysfunction plays a role in its pathogenesis [1,2]. Its main physiopathological characteristics are infiltration of lymphomononuclear cells into the perivascular space, swelling or proliferation of endothelial cells, partial obliteration of small vessels, and fibrinoid degeneration [3]. In BD, the occurrence of various immune reactions due to vascular endothelial injury results in the development of vasculitis and endothelial dysfunction. However, the mechanisms causing vasculitis and endothelial dysfunction have not yet been fully elucidated [4]. 

Vascular endothelial cells have been reported to play significant roles in numerous significant physiological and pathological reactions such as inflammation/immunity reactions, vascular tonus, hemostasis/thrombosis, angiogenesis, bleeding, disseminated intravascular coagulation, and neovascularization [5]. It has been shown in in vitro studies that a specific T cell population named angiogenic T (Tang) cells stimulates the differentiation of endothelial progenitor cells (EPCs) and might support vasculogenesis and endothelial repair by enhancing endothelial cell proliferation via vascular endothelial growth factor (VEGF) and various cytokines [6]. EPCs are produced in the bone marrow and mobilized to peripheral circulation to respond to various stimuli such as vascular inflammation, injury, and tissue ischemia and to differentiate into mature endothelial cells to reestablish vascular integrity blood flow [7]. EPCs have been demonstrated to circulate with CD45, CD31, CD34, CD309, and SYTO16 monoclonal antibodies [8]. In addition, Tang cells co-express CD3, platelet–endothelial cell adhesion molecule (CD31), and CXC chemokine stromal-cell-derived factor 1 (SDF-1)/(CXCL12, CXCR4 or CD184) [9]. As a subset of CD3+ T cells, Tang cells also express either CD4 or CD8. It has been reported that a decreased number of Tang cells is associated with vascular diseases [6,7].

In the limited number of studies on this subject in BD, controversial results have been reported. There are consistencies among the results of the limited number of studies investigating the relationship between disease activity and EPCs [7,10,11]. Aside from the studies reporting that decreases in circulating EPCs might be associated with endothelial dysfunction and vascular injury [7,12], some studies have reported increasing EPC counts in patients who were in the active disease period [11] or who had vascular thrombosis [10,13]. On the other hand, there is limited evidence in the literature about the relationship between Tang cells and EPCs with BD. 

This study aims to determine the numbers of circulating Tang cells and EPCs using multicolor flow cytometry in BD patients and investigate whether there is an association between disease activity and the numbers of these two cells and, moreover, whether these cell numbers can be used to diagnose the disease.

## 2. Materials and Methods

### 2.1. Patients and Method

The Physical Medicine and Rehabilitation Clinic and Medical Biochemistry Department at Ataturk University, Faculty of Medicine, conducted this study between March 2019 and August 2019. We included 50 patients aged 18–66 years with a diagnosis of BD according to the International Study Group (ISG) criteria [14] and 45 age- and gender-matched healthy controls. The same physician assessed all patients. The participants in both groups comprised individuals whose complete blood counts, particularly of white blood cells, were within the normal range. The Ethics Committee of Ataturk University Medical Faculty approved the study (4 October 2018/06; 51). Ataturk University Scientific Research Projects Coordination Unit supported our study within the context of scientific research projects (31 December 2018/TSA-2019-6950). Written consent was obtained. The study was performed following the Helsinki Declaration. Patients with a comorbid condition that can affect WBC, Tang cell, and EPC counts, such as systemic inflammatory, autoimmune, hematological, cardiovascular, or rheumatological disorders, acute or chronic infection, malignancy, chronic renal failure, diabetes mellitus, cerebrovascular events, hypertension, familial hypercholesterolemia, and those with a history of pregnancy or of surgical intervention/trauma within the last three months were excluded. 

Demographic, clinical, and laboratory characteristics of the patients were collected from the participants’ medical records, and the data are shown in Table 1.

The patients’ disease activities were scored using the Turkish version of the Behçet Disease Current Activity Form (BDCAF) 2006 [15]. This form involves the assessment of only clinical features. In this form, which does not involve the pathergy test or laboratory results, every symptom manifested according to the involved system is scored based on its duration within the last four weeks and then evaluated. Patients are scored between 0 and 1 point according to the presence of symptoms such as headache, oral ulcers, genital ulcers, cutaneous lesions such as erythema and pustules, articular features such as arthritis and arthralgia, and signs of gastrointestinal symptoms such as nausea, vomiting, and abdominal pain/diarrhea, or bloody stools within the last four weeks. Moreover, patients are questioned regarding symptoms involving large vessels or the nervous system and eyes, which is scored between 0 and 7 according to new symptom development within the last four weeks. The total score range is 0–12 points. Patients with a BDCAF index score below 2 are considered inactive, and those with a score equal to or above 2 have active disease [16].

While taking samples, the patients were questioned about their drug use history, and samples were obtained when no drug was taken. Samples for flow cytometry analysis were taken after obtaining samples for complete blood count to avoid endothelial cell contamination due to phlebotomy. Whole blood samples were taken into hemogram tubes containing di-potassium ethylenediaminetetraacetic acid (K_2_-EDTA) (Becton Dickinson-BD, San Diego, CA, USA) and transferred to the laboratory within two hours for analysis. Analyses for complete blood count were conducted on the Sysmex XN-1000 Hematology Analyzer (Sysmex Corporation, Kobe, Japan).

### 2.2. Flow Cytometry Analysis

Analysis of circulating Tang cells in fresh whole blood was conducted using a flow cytometry device. For analysis, CD45 peridinin chlorophyll protein-cyanine dye 5-5 (PerCP-Cy5-5), CD3 fluorescein isothiocyanate (FITC), CD31 phycoerythrin (PE), CD184 allophycocyanin (APC), CD4 allophycocyanin cyanine dye 7 (APC-Cy5-5), and CD8 phycoerythrin cyanine 7 (PE-Cy7) monoclonal antibody (moAb) panel and isotype controls (BD Biosciences, San Diego, CA, USA) were used.

Circulating EPCs were analyzed using CD45 peridinin chlorophyll protein-cyanine dye 5-5 (PerCP-Cy5-5), CD31 phycoerythrin (PE), CD34 phycoerythrin cyanine 7 (PE-Cy7), nuclear staining SYTO16 FITC (Dako, Germany), and CD309 allophycocyanin (APC) monoclonal antibody (moAb) panel and isotype controls (BD Biosciences, San Diego, CA, USA), following the analysis steps according to the manufacturer’s protocols.

Analysis of circulating Tang cells and circulating EPCs in fresh whole blood were conducted using a flow cytometry device (FACS Diva software (BD Biosciences)) according to the method suggested by Manetti et al. [9] and Falay et al. [8].

**Figure 1:**
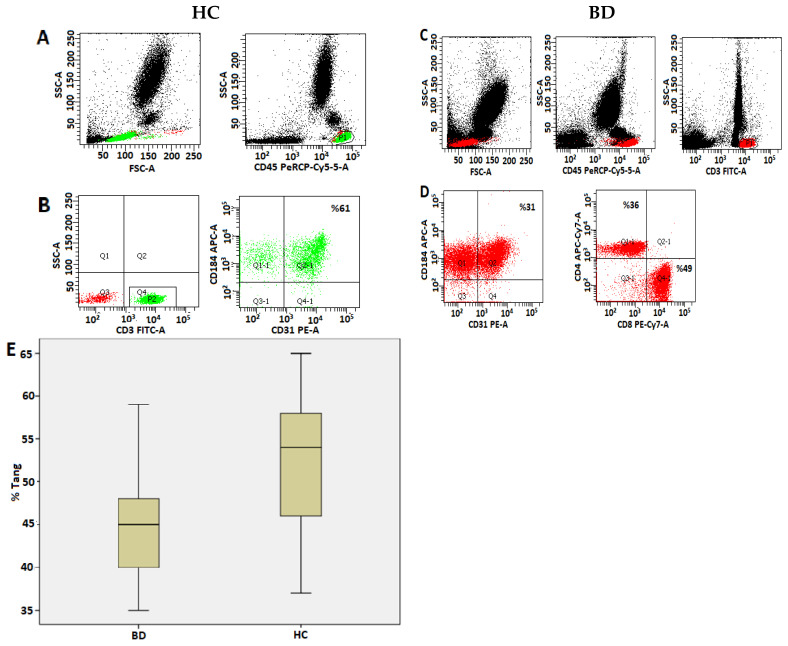
Peripheral blood Tang cell analysis histogram samples in patients with Behçet disease and healthy controls and the comparison chart. Gating strategy used for the flow cytometric identification of Tang cells: the percentage of Tang cells was determined as the percentage of CD3+ CD31+ CD184+ common positive cells by gating CD3-positive T lymphocytes. Quadrant graphs adjusted for fluorescence signals provided by isotype controls. (**A**,**C**): Lymphocytes were gated with the CD45/SSC histogram. (**B**,**D**): CD3-positive T lymphocytes gated (yellow cell population in A and red cell population in (**C**)). CD3-positive T lymphocytes were gated and the percentage of CD31/CD184 co-positive cells were determined. (**E**): Box plot of percentages of circulating CD3 + CD31 + CD184+ Tang cells in total CD3+ T cells for HC and BD patients.

Gating strategy for Tang cells: Lymphocytes were gated from the CD45/SSC histogram. The percentages of CD31/CD184 co-positive cells and the circulating Tang cells were determined by gating the T cells with the CD3/SSC histogram (Figure 1).

**Figure 2:**
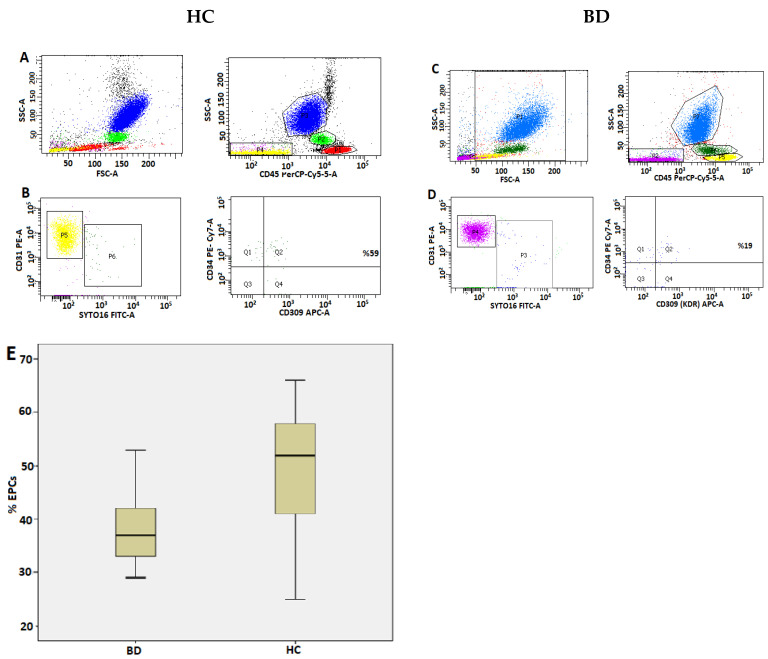
Peripheral blood EPC analysis histogram samples in patients with Behçet disease and healthy controls and the comparison chart. Gating strategy used for the flow cytometric enumeration of EPCs. (**A**,**C**): Hematopoietic cells were excluded by CD45/SS (yellow cell population in A and pink cell population in (**C**)). (**B**,**D**): Platelets excluded with CD31/SYTO16. SYTO16-positive cells were gated and the percentage of CD34/CD309 co-positive cells were determined. Quadrant graphs adjusted for fluorescence signals provided by isotype controls. (**E**): Box plot of percentages of circulating SYTO16+ CD34+ CD309+ EPCs in HC and BD patients.

Gating strategy for EPCs: Hematopoietic cells were excluded by the CD45/SSC histogram. Nucleated cells were gated with the DNA marker SYTO16, excluding CD31-positive platelets from the CD45-negative area. The percentage of EPCs was determined based on the CD34 and CD309 (VEGFR-2/KDR) co-positive cells in this field (Figure 2). The absolute numbers of both cell populations were calculated based on the percentages of these cell populations determined by flow cytometry and the WBC count obtained from the complete blood count device (Sysmex XN-1000 Hematology Analyzer (Sysmex Corporation)) (formulas: Tang cell% × WBC count/100 and EPC% × WBC count/100).

### 2.3. Statistical Analysis

Statistical analysis was performed using SPSS 20.0 (SPSS, Chicago, IL, USA) software. The Kolmogorov–Smirnov test was used to assess the conformity of the parameters to the normal distribution. Independent sample *t*-tests (an independent test and a Student *t*-test) and the Mann–Whitney U test were used to compare the patient and control values. Pearson correlation analysis was used to assess correlations among the parameters. The results are presented as the mean ± standard deviation (SD) and median with the IQR. *p* < 0.05 is the threshold used for determining significant differences between the groups.

## 3. Results

In the study, 50 patients (24 females and 26 males) with a mean age of 37 ± 10 years, ranging from 18 to 66 years, diagnosed with BD and 45 age- and gender-matched healthy controls (24 females and 21 males) were included. Of the patients, 44 were using colchicine, 1 was using hydroxychloroquine, 16 were using azathioprine, 10 were using tumor necrosis factor-alpha inhibitor, 1 was using mycophenolate mofetil, 1 was using sulfasalazine, 2 were using cyclosporine, and 11 were using steroids. Among the patients with active disease, 14 were receiving colchicine only, 3 were using colchicine and at least one immunosuppressive treatment, and 1 was using azathioprine and anti-TNF therapy. On the other hand, 14 of the patients with inactive disease were receiving colchicine, 13 were receiving colchicine and immunosuppressive therapy, and the others were receiving immunosuppressive therapy without colchicine treatment.

Among the patients, Tang cell count, Tang cell%, EPC count, and EPC% were found to be statistically insignificant between the patients using immunosuppressive drugs and those not using immunosuppressive drugs (*p* > 0.05). Moreover, between the patients using anti-TNF drugs and those not using anti-TNF drugs, differences in Tang cell count, Tang cell%, EPC count, and EPC% were found to be statistically insignificant (*p* > 0.05).

The demographic, clinical, and laboratory characteristics of the patients and healthy controls are presented in Table 1. There were no differences between the two groups regarding demographic characteristics, complete blood count, lymphocyte subtypes, and laboratory parameters (*p* > 0.05). However, the circulating Tang cell and EPC counts and percentage values were determined to be significantly lower in the BD patient group than in the control group (*p* < 0.05) (Table 1). The histogram analysis regarding samples of Tang cells and EPCs in the peripheral blood and the comparison graphs of the groups’ percentage values are shown in Figure 1 and Figure 2, respectively. The average disease duration in the BD patient group was 100 ± 81.7 months. The patients’ mean BDCAF score was 3.5 ± 1.9 (Table 1).

The data regarding the comparisons of complete blood count parameters, lymphocyte subtypes, and Tang cell and EPC counts between inactive (n = 18) and active (n = 32) patients, grouped according to the clinical parameters of BDCAF, are presented in Table 2. While the percentages of Tang cells and EPCs in active BD patients were significantly lower (*p* < 0.05), there was no statistical difference between the two groups (active and inactive BD patients) regarding the absolute cell count (Table 2).

Table 3 summarizes organ involvement and the frequency and percentage of BD Current Activity Form 2006 parameters in patients with BD.

**Figure 3:**
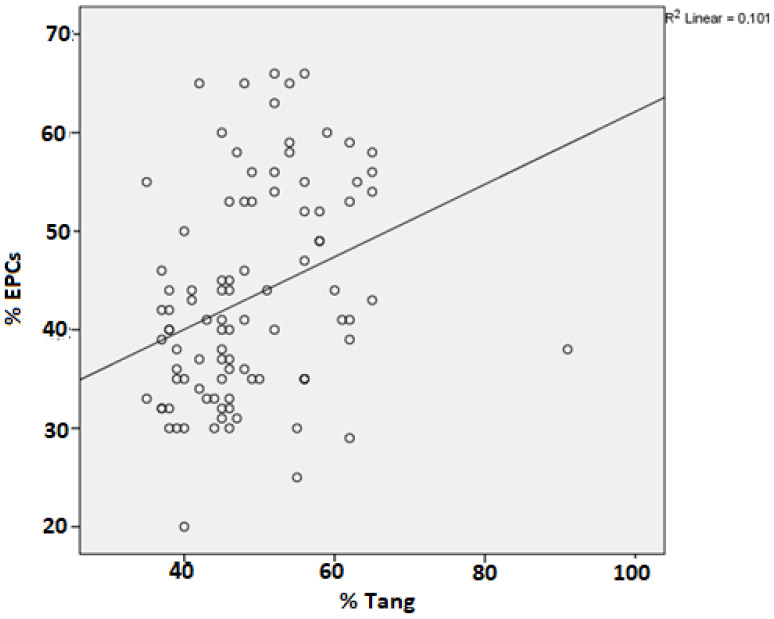
Correlation between the circulating Tang cells and EPCs in patients with Behçet disease.

A weak significant positive correlation was present between Tang cell and EPC percentage values in the patients with BD (r: 0.318, *p* = 0.002) (Figure 3).

## 4. Discussion

In our study, the circulating Tang cell and EPC counts were significantly lower in the BD patients than in the healthy controls. The active BD patients had significantly lower blood Tang cell and EPC percentage values than the inactive BD patients. Moreover, there was a weak significant positive correlation between the blood Tang cell and EPC counts in BD. It is known that in healthy patients, EPCs participate in the repair process when there is a vascular injury. Recent data also suggest that Tang cells have a role in vascular repair. In this situation, EPCs are mobilized from the bone marrow and can be detected in the circulation. [6,17,18,19]. There are similar results in the literature. Low EPC counts, reflecting diminished vascular repair, have been reported to be associated with cardiovascular morbidity and increased mortality [20]. These results may not be BD-specific. Vascular inflammation in any setting may cause findings similar to the cases in our study. BD is characterized by chronic vascular inflammation, and Tang cell and EPC levels are decreased in the active disease state. However, in this study, we aimed to determine the correlations between disease activity and Tang cell and EPC counts, and we conclude that there is a correlation. Furthermore, these results show that using Tang cells and EPCs as disease activity markers is possible.

Since the absolute number is affected by the WBC count, there was a difference in Tang cell and EPC percentage values between the active and inactive BD patients but not in absolute counts, which were calculated using total WBC count and percentage values.

BD typically targets small or large arteries and veins. The progression of vascular disease may result in arterial obstruction, venous thrombosis, and aneurysms in addition to systemic but non-specific symptoms such as fever, myalgia, and joint pain. Such inflammatory situations might have originated from an autoimmune reaction against autoantigens in the vessel wall, which are currently unidentified [21]. Tang cells, a subset of T cells with crucial immunological functions and constituting the central cluster of EPC colonies in human peripheral blood mononuclear cell cultures, are cells with proangiogenic functions capable of endothelial repair through interacting with EPCs and endothelial cells. It has also been shown that Tang cell reduction can hinder EPC differentiation and functionality [6].

According to the literature, the EPC count increases with acute inflammation [9]. The cause of acute-inflammation-related increases in EPC values might be the increase in Tang cells. The mean ESR and CRP values, which are among the acute inflammation indicators, were normal or very close to normal and similar in both groups, which might reveal that our results were unaffected or minimally affected by acute inflammation. Therefore, we may consider that the Tang cell pool might have been reduced due to the continuity of a chronic inflammatory disorder such as BD. On the other hand, it may also reveal that the Tang cell pool reduction might have caused an insufficient immune response against chronic inflammation, and thus, decreased Tang cell counts might play a role in disease activation.

EPCs are mobilized from the bone marrow to the peripheral circulation to respond to situations causing vascular injury [2]. EPCs play a role in both physiological and pathological processes [4]. In experimental and human studies, reduction in or dysfunction of EPCs has been reported to occur in vascular disorders associated with endothelial dysfunction and reduced vascular wall reparation capability after endothelial injury and damage [20,22,23]. However, the number of studies on EPCs in BD are limited, and the information they report is controversial.

In the study by Fadini et al. [7], the EPC count was reported to decrease in BD patients compared with healthy controls. In that study, it was stated that the chronicity of the disease might have led to long-term progressive EPC depletion. In addition, it was expressed that the reduction in EPCs due to the irregularity of vascular homeostasis might have been both a triggering factor for the autoreactive immune response and a factor in increased tissue damage. In the study by Bozkırlı et al. [10], lower EPC counts were determined in BD patients than in healthy individuals, even though the difference was statistically insignificant. In the study, it was stated that the EPC count was not related to disease activity but was higher in patients with no acute inflammation but with thrombosis. As a result, they reported that thrombosis or vascular injuries were the main determinants of EPCs, independent of underlying diseases.

However, in the study by Arıca et al. [11], the EPC count was higher in BD patients than in healthy controls. Moreover, the EPC count was higher in patients with active disease than those with inactive disease. Furthermore, in the same study, the acute inflammation indicators such as the CRP and ESR values were significantly higher in the BD patient group than in the control group. Thus, the conclusion of that study was that EPC count might increase during exacerbations of vascular inflammation, and while acute inflammation increases the EPC count, a progressive reduction in EPCs might accompany chronic inflammation in the long term.

In our study, the mean ESR and CRP values, being normal or very close to normal and similar in both groups, might let us consider that our results were not affected by acute inflammation. Moreover, while the disease durations of the three abovementioned studies were 6, 5, and 5.5 years, respectively [7,11], the disease duration was approximately 8.5 years in our study. This might demonstrate that chronic inflammation can cause progressive depletion of EPCs in the long term, as in the other three studies. Low Tang cell and EPC counts might remove the protective masks of the structures ordinarily unexposed to the immune system and give way to chronic inflammation by causing the emergence of autoreactive T cells. Moreover, a low EPC level might prevent endothelial repair and reformation of a healthy intimal layer and accelerate injury progression in the later stages of vascular damage formation. This model is also supported by the low EPC counts in patients with ANCA-related vasculitis [24,25]. Rouhl et al. [26] found lower Tang cell numbers in hypertensive patients with cerebral small vessel disease, independent of age. Therefore, this may suggest that low Tang cell and EPC counts can play a role in disease reactivation and the failure to develop an effective response against chronic inflammation.

Even though a correlation between Tang cells and EPCs was present in our study, its cross-sectional nature does not reveal any definitive result regarding the cause–effect relationship in disease development and progression. The fact that the participating patients were receiving various immunosuppressive treatments and that some immunosuppressive drugs might have affected EPC counts represent limitations of our study.

## 5. Conclusions

In conclusion, it was determined that circulating Tang cell and EPC counts are lower in BD, and such reductions become even more profound with increasing disease activity. This may prevent the development of a sufficient immune response against a disease with a course of chronic inflammation or may trigger the formation of autoreactive immunity. A reduction in Tang cells and EPCs may serve as a marker or predictor of vascular damage in BD patients and represents the progression of vascular injury. We suggest that further studies on this subject are required, and we expect that our results should be supported by studies conducted in the future.

## Figures and Tables

**Table 1:** Comparison of demographic, clinical, and laboratory characteristics of patients with Behçet disease and healthy controls.

	Patients (n = 50) (Meanv ± vSD)	Controls (n = 45) (Mean ± SD)	*p*	95% CI
Sex (f/m)	24/26	24/21	0.823	15.67–8.32
Age (mean ± SD)	37 ± 10.7	37.2 ± 11.4	0.930	−6.41–5.96
BMI (kg/m^2^)	24.6 ± 4.1	23.5 ± 1.8	0.106	1.99–−6.02
Disease duration (median with İQR) (month)	78 (27–153)	-	-	77.3–123.73
WBC (×10^3^/μL)	8.1 ± 1.9	8 ± 1	0.747	−0.38–1.01
Neutrophile (%)	58.6 ± 10.9	59.6 ± 9	0.607	−5.12–3.01
Lymphocyte (%)	31.2 ± 10.1	30.7 ± 8.5	0.790	−3.31–4.34
Monocyte (%)	8 ± 2	8 ± 2.8	0.788	−3.27–4.30
ESR (median with İQR) (min–max) (mm/h)	7 (5–19) (1–54)	10 (4–14) (2–20)	0.762	−3–3
CRP (median with İQR) (min–max) (mg/mL)	3 (3–3.85) (3–55)	3 (2–5) (2–20)	0.368	0.0–1
CD3 (%)	72.3 ± 7	73.3 ± 7.9	0.501	−4.08–2
CD4 (%) (in total lyphocytes)	54 ± 10	58.3± 7.5	0.022	−7.91–0.62
CD8 (%) (in total lyphocytes)	31.8 ± 6.7	33.8 ± 6	0.119	−4.66–0.54
Tang cell count (cells/μL)	3.5 ± 1.2	4 ± 0.9	0.046 *	−0.88–−0.007
EPC count (cells/μL)	2.9 ± 0.9	3.7 ± 1	0.001 **	−1.19–−0.38
Tang cells (%)	44.6 ± 5.4	52.4 ± 8	0.001 **	11.71–4.86
EPCs (%)	37.6 ± 6.8	49.5 ± 10.9	0.001 **	15.67–8.32
BDCAF score	3.5 ± 1.9	-	-	-

f: female; m: male; SD, standard deviation; BMI: body mass index; IQR: interquartile range; WBC: white blood cell; MPV: mean platelet volume; PDW: platelet distribution width; PCT: plateletcrit; NLO: neutrophil/lymphocyte ratio; PLO: platelet/lymphocyte ratio; ESR: erythrocyte sedimentation rate; CRP: C-reactive protein; EPCs: endothelial progenitor cells; BDCAF; Behçet Disease Current Activity Form 2006. * *p* < 0.05: statistically significant difference between groups; ** *p* < 0.01: statistically significant difference between groups. CI: 95% confidence interval of the difference.

**Table 2:** Comparison of complete blood count parameters, lymphocyte subtypes, and Tang cell and EPC levels in patients with active and inactive Behçet disease.

	Inactive (n: 18) (Mean ± SD)	Active (n: 32) (Mean ± SD)	*p*	95% CI
WBC (×10^3^/μL)	7.96 ± 2.4	7.75 ± 2.1	0.747	−1.08–1.49
Neutrophile (%)	59.8 ± 13.2	57.9 ± 9.3	0.549	−4.50–8.36
Lymphocyte (%)	30.5 ± 12.6	31.6 ± 8.6	0.712	−7.15–4.92
Monocyte (%)	8 ± 1.4	8.2 ± 2.1	0.702	−1.33–0.90
ESR (median with IQR) (mm/h)	6 (5–23.75)	7 (4.25–17.25)	0.7	−3.0–5
CRP (median with IQR) (mg/mL)	3 (3–3)	3 (3–4)	0.119	0.0–0.0
CD3 (%)	74.7 ± 5.3	70.9 ± 7.6	0.07	−0.32–7.83
CD4 (%)	54.5 ± 10.4	53.7 ± 9.9	0.795	−5.22–6.79
CD8 (%)	31.5 ± 5.6	31.9 ± 7.3	0.874	−4.33–3.70
Tang cell count (cells/μL)	3.9 ± 1.4	3.3 ± 1	0.099	−0.11–1.28
EPC count (cells/μL)	3.2 ± 1	2.8 ± 0.8	0.074	−0.05–1.01
Tang cells (%)	48.9 ± 7.9	42.5 ± 4.9	0.001 **	2.75–10.03
EPCs (%)	41.2 ± 6.3	35.5 ± 6.4	0.004 **	1.88–9.39
BDCAF score	1.67 ± 0.49	4.56 ± 1.54	0.001 **	−3.65–−2.14

SD: standard deviation; WBC: white blood cell; IQR: interquartile range ** *p* < 0.01: statistically significant difference between groups, CI: 95% confidence interval of the difference.

**Table 3:** The frequency and percentage of Behçet Disease Current Activity Form-2006 parameters in patients with Behçet disease.

Clinical Symptoms and Signs		With; n (%)	Without; n (%)	*p*
Headache		34 (68%)	16 (32%)	0.027 *
Oral ulcer		29 (58%)	21 (42%)	0.943
Genital ulcer		8 (16%)	42 (84%)	0.460
Skin lesions	Erythema	18 (36%)	32 (64%)	0.987
	Pustule	24 (48%)	26 (52%)	0.912
Joint involvement	Arthralgia	38 (76%)	12 (24%)	0.175
	Arthritis	5 (10%)	45 (90%)	0.630
GIS involvement	Abdominal pain	13 (26%)	37 (74%)	0.246
	Bloody diarrhea	2 (4%)	48 (96%)	-
Eye involvement (Active uveitis)		1 (3%)	49 (98%)	-
Nervous system involvement		2 (4%)	48 (96%)	-
Great vascular involvement		-	50 (100%)	-

n: number of patients with Behcet’s disease with symptoms or signs, %: percentile, GIS: gastrointestinal system; * *p* < 0.05: statistically significant difference between groups.

## Data Availability

Not applicable.
